# A Lattice Model for Elastic Particulate Composites

**DOI:** 10.3390/ma11091584

**Published:** 2018-09-01

**Authors:** Darius Zabulionis, Vytautas Rimša

**Affiliations:** 1 Laboratory of Experimental Mechanics, Institute of Mechanical Science, Vilnius Gediminas Technical University, Vilnius 10221, Lithuania; 2 Department of Aviation Technologies, Antanas Gustaitis’ Aviation Institute, Vilnius Gediminas Technical University, Vilnius 10221, Lithuania; vytautas.rimsa@vgtu.lt

**Keywords:** particulate composite, bonded particles, lattice model, spring network model, discrete element method

## Abstract

In the present article, a version of the lattice or spring network method is proposed to model the mechanical response of elastic particulate composites with a high volume fraction of spherical particles and with a much weaker matrix compared to the stiffness of the particles. The main subject of the article is the determination of the axial stiffnesses of the springs of the cell. A comparison of the mechanical response of a three-dimensional particulate composite cube obtained using the finite element method and the proposed methodology showed that the efficiency of the proposed methodology increases with an increasing volume fraction of the particles.

## 1. Introduction

The lattice or spring network method is applied widely in various areas of mechanics: to solve various problems of continuum mechanics, micromechanics, molecular dynamics, fracture mechanics, multiscale modelling, soft materials, and so on [1,2,3,4,5,6]. Apart from that, this method can also be applied to model different materials: metals, concrete, asphalt, ceramics, various composites, particulate solids, granular matter, and biomaterials [7,8,9,10]. In addition, the lattice method can be applied to elasticity and viscoelasticity problems [1], or used as an alternative to the finite element method [2].

The geometry of a cell that approximates a continuum, the model of the spring of the cell, and the determination of the required stiffness parameters of the spring are three of the most important issues in the lattice model. When springs are used as the connecting elements of the nodes of the cells, only one parameter of the springs, i.e., the axial stiffness, has to be determined in terms of the material properties. It is relatively easier to determine the stiffness parameters of the spring for homogeneous materials than for composites. One approach to model composites using the lattice model is to approximate the constituent parts of the composites—for example, the matrix and the particles—by distinct springs for each constituent part of the composite [8,11,12]. In [12], it is assumed that the composite, consisting of bonded spherical particles, is approximated by a network of one-dimensional springs connecting the centres of the interacting spherical particles. The total stiffness of the spring is calculated as the total stiffness of the three distinct springs concatenated in a series. Two springs, the first and third, correspond to the interacting particles, while the intermediate spring corresponds to the bond between the particles. A similar approach was proposed in [13]. In this article, the convex spherical surfaces of the particles and the concave spherical surfaces of the bond element were taken into account in the evaluation of the axial stiffness of the spring that connects the centres of the two interacting particles. In this article, the connecting spring was also modelled as three distinct springs concatenated in series.

In the present paper, the ideas of the evaluation of the stiffness of the spring that connects the centres of two interacting adjacent particles, presented in article [13], are extended by evaluating the lower and upper bounds of the stiffness of the spring. Two different approaches were applied to evaluate the stiffnesses: the conditional connecting element was divided into infinitely small prisms, and infinitely thin rings. The former provides the lower bound and the latter provides the upper bound. The obtained closed-form solutions of both bounds of the stiffnesses were verified using the finite element method by modelling a 3D elastic particulate composite with different particle volume fractions, different Poisson’s ratios, and different ratios of the modulus of elasticity of the particles to the matrix.

**Figure 1:**
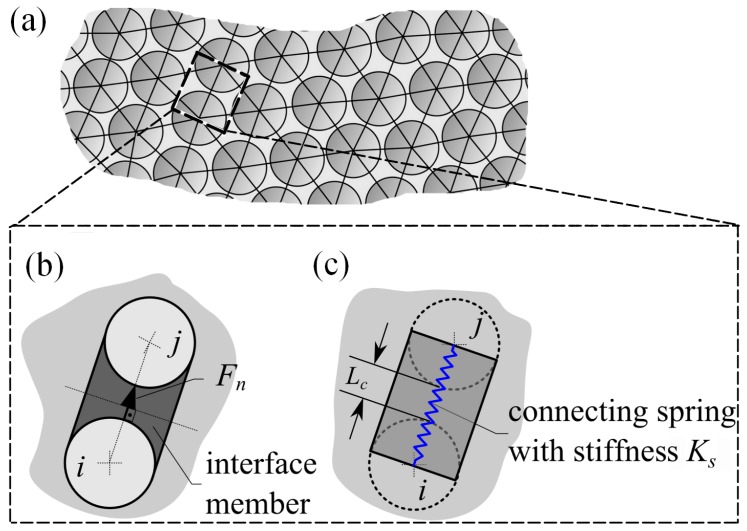
Modeling concept: a particulate composite with the conditional connecting element (**a**); a normal interaction of two spheres through a conditional interface member (**b**); and a spring representing the interaction of the spheres (**c**).

A particulate composite consisting of particles embedded in the matrix is approximated by one-dimensional springs that connect the centres of the particles (Figure 1). The springs are characterized by their length $R_{p}+L_{c}$, where $R_{p}$ is the particle diameter and $L_{c}$ is the distance between the particles, and the axial stiffness is $K_{s}$ (Figure 1b).

The following assumptions are valid for the particulate composite and its approximating spring network:

- the particles, matrix and the connecting springs obey the linear elastic law,
- the particles interact with the matrix by their entire surface,
- the particles of the composite do not rotate,
- the interface member connecting two adjacent particles is composed of the matrix and is cylindrical,
- the diameters of the ends of the interface member are the same as of connected particles,
- the interface members interact only with the two adjacent particles and interact with the particles only by the entire surface of the hemispheres,
- the pin-connected springs connect the centres of the adjacent particles,
- the spring is composed of the interface member and two hemispheres, and only carries an axial force.

All quantities related to the particles are denoted by the subscript *p* and the quantities related to the interface members are denoted by the subscript *b*. The particle is characterised by the radius $R_{p}$, elasticity modulus $E_{p}$, and Poisson’s ratio $v_{p}$. The interface member is characterised by the cylinder radius $R_{b}$ and the elasticity constants $E_{m}$ and $v_{b}$, respectively. Two limits $K_{s,I}$ and $K_{s,II}$ of the axial stiffness can be obtained by considering two different divisions of the connecting element: as parallel and sequentially connected springs (Figure 1).

### 1.1. Governing Equations for the Lower Bound of the Stiffness

**Figure 2:**
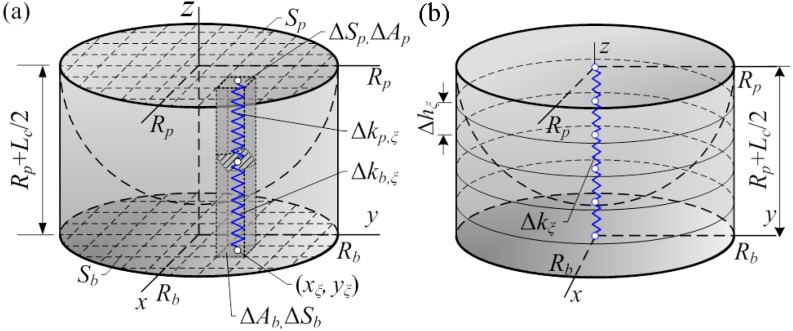
Concepts of the discretisation of a half of the conditional connecting elements: by parallel connected prisms (**a**) and by sequentially connected rings (**b**).

Let us consider the half of the connecting element comprised of a half of the particle and a half of the interface member to obtain the lower bound $K_{s,I}$ of the stiffness of the connecting element (Figure 2). The entire hemisphere is denoted hereafter by $\Omega_{p}$ and its circular basis by $S_{p}$. The entire half of the interface member is denoted hereafter by $\Omega_{b}$ and its circular basis by $S_{b}$.

Let the circular bases $S_{p}$ and $S_{b}$ of the interface member and the hemisphere (Figure 2a) be divided into *n* infinitesimal rectangles $S_{p,\xi }$ and $S_{b,\xi }$ so that $S_{i}={⋃}_{\xi =1}^{n}S_{i,\xi }$ and ${⋂}_{\xi =1}^{n}S_{i,\xi }=⌀$, where i∈{p,b}. Then, the areas $\Delta A_{i,\xi }$ of the rectangles $S_{i,\xi },i\in \left\{p,b\right\}$, are such that $A_{i}=\sum_{\xi =1}^{n}\Delta A_{i,\xi }$, where $A_{i},i\in \left\{p,b\right\}$ is the total area of the circular basis $S_{i}$. Let the hemisphere $\Omega_{p}$ and the interface member $\Omega_{b}$ (Figure 1a) be divided into infinitesimal parallel connected prisms $\Delta \Omega_{i,\xi }$ whose bases are the rectangles $S_{i,\xi }$ so that $\Omega_{i}={⋃}_{\xi =1}^{n}\Omega_{i,\xi }$, i∈{p,b}; see Figure 2a.

Then, the stiffness of the prisms $\Omega_{p,\xi }$ and $\Omega_{b,\xi }$ connected sequentially can be expressed as follows (in Figure 2a only half of the prisms are depicted):

(1) $$\Delta k_{\xi }=\frac{1}{2}\frac{\left(\frac{D_{p}D_{m}}{l_{p}\left(x_{p,\xi },y_{p,\xi }\right)l_{b}\left(x_{b,\xi },y_{b,\xi }\right)}\right)\Delta A_{i,\xi }}{D_{p}/l_{p}\left(x_{p,\xi },y_{p,\xi }\right)+D_{m}/l_{b}\left(x_{b,\xi },y_{b,\xi }\right)},$$

where $D_{p}$ and $D_{m}$ are the elastic constants of materials of the particle and interface member, respectively. In case of the uniaxial stress state of the particle and the interface member, i.e., when $\sigma_{xx}=\sigma_{yy}=0$ of the conditional connecting element, see Figure 2, the elastic constant $D_{p}=E_{p}$ and $D_{m}=E_{m}$. When it is assumed that, for the conditional connecting element $\epsilon_{xx}=\epsilon_{yy}=0$, then $D_{i}=E_{i}\left(1-\nu_{i}\right)/\left(\left(1+\nu_{i}\right)\left(1-2\nu_{i}\right)\right)$, where i∈{p,m}. Other cases of the elastic constants are also possible. In Equation (1), $l_{p}\left(x_{p,\xi },y_{p,\xi }\right)$ and $l_{b}\left(x_{b,\xi },y_{b,\xi }\right)$ are the half lengths of the prisms of the hemispheres $\Omega_{p,\xi }$ and $\Omega_{b,\xi }$ at the points $(x_{p,\xi },\;y_{p,\xi })\in S_{p}$ and $\left(x_{b,\xi },y_{b,\xi }\right)\in S_{b}$, respectively. The total stiffness of all prisms $\Omega_{\xi }=\Omega_{p,\xi }⋃\Omega_{b,\xi },\xi \in \left\{1,\ldots ,n\right\}$, or the stiffness of the connecting element $K_{s}=\sum_{\xi =1}^{n}\Delta k_{\xi }$. By letting $\Delta A_{i,\xi }\to 0$, we obtain a limit $K_{s,I}=\lim_{\Delta A_{i,\xi }\to 0}\sum_{\xi }\Delta k_{\xi }$, where $\Delta k_{\xi }$ is given in Equation (1). The obtained limit can be rewritten as a double integral over $S_{b}$, since $S_{b}=S_{p}$

(2) $$K_{s,I}={\;\int \int \;}_{S_{b}}T(x,y)dA,$$

where the integrand T(x,y)

(3) $$T\left(x,y\right)=\frac{1}{2}\frac{\frac{D_{p}D_{m}}{l_{p}\left(x,y\right)l_{b}\left(x,y\right)}}{\frac{D_{p}}{l_{p}\left(x,y\right)}+\frac{D_{m}}{l_{b}\left(x,y\right)}}.$$

In Equation (3), the half lengths of the prisms $l_{p}\left(x,y\right)$ and $l_{b}\left(x,y\right)$ at the point $\left(x,y\right)\in S_{b}$ are

(4) $$l_{p}\left(x,y\right)=\sqrt{R_{p}-x^{2}-y^{2}},$$

(5) $$l_{b}\left(x,y\right)=L_{c}/2+R_{p}-\sqrt{R_{p}-x^{2}-y^{2}}.$$

The integral of Equation (2) can be expressed as an iterated double integral in the rectangular coordinate system

(6) $$K_{s,I}=\int_{-R_{b}}^{R_{b}}\int_{0}^{\sqrt{R_{b}^{2}-x_{i}^{2}}}T(x,y)dxdy$$

in the polar coordinates

(7) $$K_{s,I}=\pi \overset{R_{p}}{\underset{0}{\int }}\frac{\frac{D_{p}D_{m}}{\sqrt{R_{p}^{2}-r^{2}}\left(L_{c}/2+R_{p}-\sqrt{R_{p}^{2}-r^{2}}\right)}}{\frac{D_{p}}{\sqrt{R_{p}^{2}-r^{2}}}+\frac{D_{m}}{L_{c}/2+R_{p}-\sqrt{R_{p}^{2}-r^{2}}}}r\;dr,$$

where $r=\sqrt{x^{2}+y^{2}}$. After a change of variable $z=(R_{p}^{2}-r^{2})$ in Equation (7), we obtain

(8) $$K_{s,I}=\frac{1}{2}\pi \int_{0}^{R_{p}^{2}}\frac{D_{p}D_{m}}{\left(L_{c}/2\;+R_{p}\right)D_{p}+\left(D_{m}-D_{p}\right)\sqrt{z}}dz.$$

Integration of Equation (8) yields the stiffness of the connecting element as $D_{p}\neq D_{m}$

(9) $$K_{s,I}=\frac{1}{2}\pi D_{p}D_{m}\left(2\frac{R_{p}}{b}+\frac{a}{b^{2}}\ln\left(\frac{a^{2}}{{\left(a+bR_{p}\right)}^{2}}\right)\right),$$

where $a=\left(L_{c}/2+R_{p}\right)D_{p}$, and $b=D_{m}-D_{p}$. It should be noted that, when $D_{m}=D_{p}$, then $K_{s,I}$ by Equation (9) is undefined due to the division by zero, since $b=D_{m}-D_{p}=0$. In this case, $K_{s,I}$ can be obtained from Equation (7) by letting $D_{m}=D_{p}$:

(10) $$K_{s,I}=\frac{1}{2}\frac{\pi D_{p}R_{p}^{2}}{\left(L_{c}/2\;+R_{p}\right)}\;\mathrm{as}\;D_{p}=D_{m}.$$

It is easy to notice that the obtained limit of the stiffness $K_{s,I}$ in Equation (10) corresponds to the stiffness of the homogeneous cylinder, i.e., $K_{s,I}=AD/l=\pi R_{p}^{2}D/\left(2R_{p}+L_{c}\right)$, where *A* is the area of the cross-section of the cylinder, and *D* is the elastic constant of the cylinder.

### 1.2. Governing Equations for the Upper Bound of the Stiffness

In this subsection, the upper limit $K_{s,II}$ of the stiffness of the connecting element is obtained by dividing the hemisphere $\Omega_{p}$ and the interface member $\Omega_{b}$ (Figure 1b) into sequentially connected cylinders of the infinitesimal height Δh. The stiffness of the sequentially connecting cylinders can be expressed as follows:

(11) $$K_{s,II}=\frac{1}{\sum_{i}\left(1/\Delta k_{i}\right)},$$

where $\Delta k_{i}$ is the stiffness of cylinder *i* of infinitesimal height Δh

(12) $$\Delta k_{i}=\frac{A_{p}\left(z\right)D_{p}+A_{b}\left(z\right)D_{m}}{\Delta h_{i}},\;\;\mathrm{as}\;\;z\in \left[0,R_{p}\right],$$

(13) $$\Delta k_{i}=\pi D_{m}R_{p}^{2}/L_{c}\;,\;\;\mathrm{as}\;\;z\in \left[R_{p},R_{p}+\frac{L_{c}}{2}\right].$$

In Equations (12) and (13), $A_{p}\left(z\right)$ and $A_{b}\left(z\right)$ are the areas of the cross-sections of the particle and the interface member, respectively, dependent on coordinate *z*: $A_{p}\left(z\right)=\pi \left(R_{p}^{2}-z^{2}\right)$ and $A_{b}\left(z\right)=\pi z^{2}$. Then, Equation (11) can be rewritten as follows:

(14) $$K_{s,II}=\frac{1}{\frac{L_{c}}{\pi D_{p}R_{p}^{2}}+\sum_{i}\frac{\Delta h_{i}}{A_{p}\left(z\right)D_{p}+A_{b}\left(z\right)D_{m}}}.$$

The limit of the sum $\lim_{\max\left\{\Delta h_{i}\right\}\to 0}\;\sum_{i}\frac{\Delta h_{i}}{A_{p}\left(z\right)D_{p}+A_{b}\left(z\right)D_{m}}$ of Equation (14) can be written as an integral

(15) $$I=2\int_{0}^{R_{p}}\frac{dz}{\pi \left(R_{p}^{2}-z^{2}\right)D_{p}+\pi D_{m}z^{2}}.$$

Integration of Equation (15) depends on $D_{p}$ and $D_{m}$

(16) $$I=\frac{2}{\pi }\frac{\arctan\left(\sqrt{D_{m}/D_{p}-1}\right)}{\sqrt{D_{p}R_{p}^{2}\left(D_{m}-D_{p}\right)}}\;\mathrm{as}\;D_{m}>D_{p},$$

(17) $$I=\frac{2}{\pi }\frac{\mathrm{arctanh}\left(\sqrt{1-D_{m}/D_{p}}\right)}{\sqrt{D_{p}R_{p}^{2}\left(D_{p}-D_{m}\right)}}\;\mathrm{as}\;D_{p}>D_{m},$$

where the arctan and arctanh are the inverse tangent and inverse hyperbolic tangent, respectively. When $D_{p}=D_{m}$, then it is not possible to use Equations (16) and (17), since $D_{m}-D_{p}=0$. In this case, $K_{s,II}$ can be obtained from Equation (15) by letting $D_{m}=D_{p}$

(18) $$I=\frac{2}{\pi D_{p}R_{p}}\;\mathrm{as}\;D_{p}=D_{m}.$$

Finally, the stiffness $K_{s,II}$ is

(19) $$K_{s,II}=\frac{1}{L_{c}/\left(\pi D_{p}R_{p}^{2}\right)+I}.$$

It is easy to see that, when $D_{m}=D_{p}$, Equations (10) and (19) are equal, i.e., $K_{s,I}=K_{s,II}$.

### 1.3. Mathematical Analysis of the Stiffnesses

In this subsection, hereafter, an analysis of the obtained Equations (9), (10), and (19) is presented when $D_{p},D_{m}\to \infty ,\;D_{p},D_{m}\to 0,\;L_{c}\to \infty ,\;\mathrm{and}\;L_{c}\to 0$.

The stiffnesses $K_{s,I}$ and $K_{s,II}$ are nonlinear with respect to $D_{p}$, $D_{m}$, $L_{c}$ and $R_{p}$, where the nonlinearity of a function *f* is defined as $f\left(\lambda x_{1}+\lambda x_{2}\right)\neq f\left(\lambda x_{1}\right)+f\left(\lambda x_{2}\right)$, where λ is a real number.

When $D_{p}\to \infty$, then

$$\lim_{D_{p}\to \infty }K_{s,I}=\frac{1}{2}\pi D_{m}c\ln\left(\frac{c^{2}}{c^{2}-2R_{p}c+R_{p}}\right)-\pi D_{m}R_{p},$$

where $c=L_{c}/2+R_{p}$, and $\lim_{D_{p}\to \infty }K_{s,II}=\pi D_{m}R_{p}^{2}/L_{c}$. When $D_{m}\to \infty$, then $\lim_{D_{m}\to \infty }K_{s,I}=0.5\pi D_{p}R_{p}$, and $\lim_{D_{m}\to \infty }K_{s,II}=\infty$. It is evident that, when both $D_{p}$ and $D_{m}$ tend to ∞, then $K_{s,I}$ and $K_{s,II}$ tend to ∞ as well. In addition, when $D_{p}\;\mathrm{or}\;D_{m}\to 0$, then $K_{s,I}\;\mathrm{and}\;K_{s,II}\to 0$.

The limits $\lim_{L_{c}\to 0}K_{s,I}$ and $\lim_{L_{c}\to 0}K_{s,II}$ are as follows:

$$\lim_{L_{c}\to 0}K_{s,I}=\frac{1}{2}\pi D_{p}D_{m}\left(2\frac{R_{p}}{b}+\frac{R_{p}D_{p}}{b^{2}}\ln\left(\frac{{\left(R_{p}D_{p}\right)}^{2}}{{\left(R_{p}D_{p}+bR_{p}\right)}^{2}}\right)\right),$$

where *b* is given in the explanations of the notations below Equation (9), and $\lim_{L_{c}\to 0}K_{s,II}=1/I$, where *I* is given in Equations (16)–(18). When $L_{c}\to \infty$, then both $K_{s,I}\to 0$ and $K_{s,II}\to 0$.

**Figure 3:**
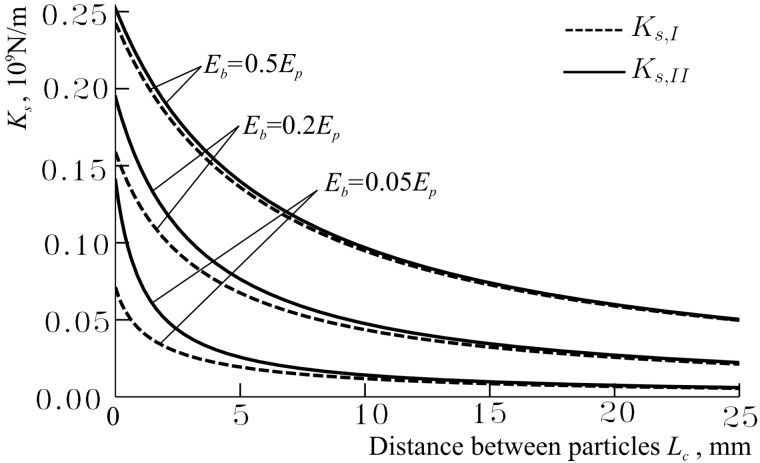
Dependences of the stiffnesses $K_{s,I}$ and $K_{s,II}$ on the distance between particles’ surfaces $L_{c}$ at different modulus of elasticity of interface member $E_{m}$ as $D_{p}=E_{p}=40$ GPa, $R_{p}=5$ mm, $D_{m}=E_{m}\in \left\{0.05E_{p},0.2E_{p},0.5E_{p}\right\}$.

It should be noted that $K_{s,II}$ tends to $K_{s,I}$ with increasing $L_{c}$ (see Figure 3); therefore, at the big values of $L_{c}$, the stiffnesses $K_{s,I}\approx K_{s,II}$ (Figure 3). In Figure 3, it is also demonstrated that $K_{s,I}$ and $K_{s,II}$ approach each other when $D_{p}\to \;D_{m}$ or $D_{m}\to \;D_{p}$.

## 2. Numerical Validation of the Proposed Methodology

Two numerical validations of the proposed methodology are presented hereafter in two subsections. In the first validation, the stiffnesses $K_{s,I}$ and $K_{s,II}$ are compared with the stiffnesses of the 3D FE models of the connecting elements. In the second one, the mechanical behaviour of the particulate composite cube approximated by the spring model (SM) and modelled by 3D FE is compared. The FE analysis was performed by ANSYS 12.

### 2.1. Stiffness of the Connecting Element

**Figure 4:**
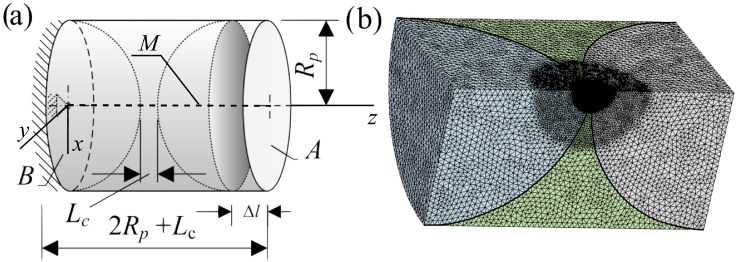
Geometry of the sample under investigation: a scheme of the connecting element (**a**), and its discretization by the tetrahedron elements “SOLID187” (**b**).

The obtained Equations (9), (10) and (19) of the stiffnesses $K_{s,I}$ and $K_{s,II}$ were verified by a 3D FE analysis of the connecting element shown in Figure 4. Two cases were considered. In the first case, $E_{p}=40$ GPa, $\nu_{p}=0.0$, $E_{m}\in \left\{40\times 10^{9},30\times 10^{9},20\times 10^{9},10\times 10^{9},5\times 10^{9},2.5\times 10^{9},5\times 10^{8},5\times 10^{7},1.0\times 10^{6}\right\}$ Pa, $\nu_{m}=0.498$. In the second case, $E_{m}=40$ GPa, $\nu_{m}=0.0$, $E_{p}\in \left\{40\times 10^{9},30\times 10^{9},20\times 10^{9},10\times 10^{9},5\times 10^{9},2.5\times 10^{9},5\times 10^{8},5\times 10^{7},1.0\times 10^{6}\right\}$ Pa, $\nu_{p}=0.498$. For both cases, $R_{p}=R_{b}=5\times 10^{-3}$ m, and $L_{c}\in \left\{5\times 10^{-6},5\times 10^{-5},5\times 10^{-4},2.5\times 10^{-3}\right\}$ m. These parameters of the particles and interface member were chosen so that it would be possible to verify the obtained equations at different moduli of elasticity $E_{i}$, Poisson’s ratios $\nu_{i},\;i\in \left\{p,m\right\}$, and at the different distances between the surfaces of the particles $L_{c}$.

The sample under investigation and its FE model, a quarter of the sample consisting of $1.087620\times 10^{6}$ nodes and $0.778623\times 10^{6}$ elements, with mesh are depicted in Figure 4a,b, respectively. The boundary conditions were as follows: $U_{z}=0$ was applied to plane *B*, and $U_{x}=U_{y}=0$ was applied to the centre line *M* (Figure 4a, dotted line) of the FE model. The FE model was discretised by the tetrahedron elements “SOLID187” of 10 nodes of six degrees of freedom (Figure 4). The average size of the finite elements of the particles of the sample was $0.01R_{p}$. The volume of the interface material was conditionally divided into two regions. The contact region between spheres was meshed by fine mesh whose average size of the finite elements was $0.015R_{p}$, while the remaining volume was meshed by a coarser mesh of an average size $0.002R_{p}$ (Figure 4b).

The stiffness obtained by the FE method, denoted hereafter as $K_{s,FEM}$, was obtained by applying a displacement Δl on free plane *A* (Figure 4a) and was calculated as $K_{s,FEM}=F/\Delta l$, where *F* is the total reaction force of plane *B* at the displacement Δl.

**Figure 5:**
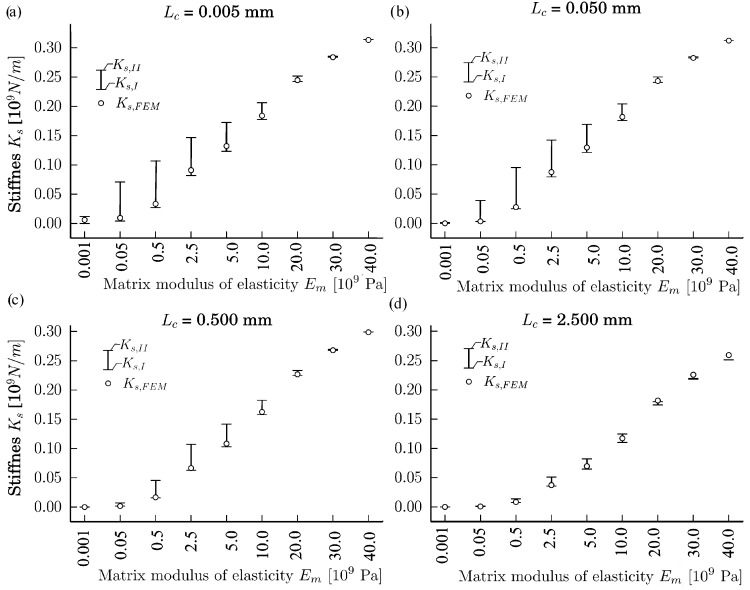
Dependences of the stiffnesses $K_{s,I}$ and $K_{s,II}$ on the modulus of elasticity of the interface member $E_{m}$ at the different distances between particles $L_{c}\in \left\{0.005,0.05,0.5,2.5\right\}$ mm: $L_{c}=0.005$ mm (**a**), $L_{c}=0.050$ mm (**b**), $L_{c}=0.500$ mm (**c**), and $L_{c}=2.500$ mm (**d**).

The calculation results are shown in Figure 5. As we can see from Figure 5, when $E_{m}\leq E_{p}$, then, in the majority of the examined cases, $K_{s,FEM}$ is closer to $K_{s,I}$ than to $K_{s,II}$, i.e., $|K_{s,I}-K_{s,FEM}\left|<\right|K_{s,II}-K_{s,FEM}|$. However, when $L_{c}=2.5\;\mathrm{mm}$ and $E_{m}\in \left\{20,30,40\right\}\;\mathrm{GPa},$ then $K_{s,FEM}$ is closer to $K_{s,II}$ than to $K_{s,I}$. In addition, from Figure 5, we can see that in the majority of cases, except from the case when $L_{c}=2.5\;\mathrm{mm}$ and $E_{m}\in \left\{20,30,40\right\}\;\mathrm{GPa}$, the stiffness $K_{s,FEM}\in \left[K_{s,I},K_{s,II}\right]$. In Figure 5, it is clearly depicted that $K_{s,I},K_{s,II}\to K_{s,FEM}$ when $E_{m}\to 0$.

### 2.2. Mechanical Behaviour of a Particulate Composite Cube

**Figure 6:**
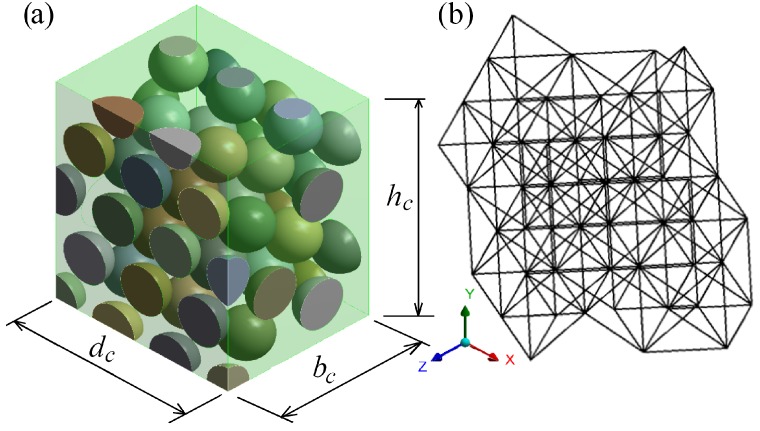
3D model of the cube particulate composite (**a**) and its spring model (**b**).

The developed SM was validated by comparing the mechanical responses of a 3D particulate composite (see Figure 6) obtained by the 3D FE method and by SM. The stiffness of the springs of SM was calculated by the developed formulas given in Equations (9) and (10).

Overall, 126 samples, which differ in the modulus of elasticity of matrix $E_{m}$ and the volume fraction of the particles $\phi_{p}$, were calculated. The properties of the 3D FE model and the SM are the following (see Figure 6): the diameters of the particles $d_{p}\in \left\{10,12,13\right\}$ mm and the corresponding volume fractions of the particles $\phi_{p}\in \left\{28.46,51.45,61.35\right\}$ %; the dimensions of the cube are (see Figure 6): height $h_{c}=40.2$ mm, width $b_{c}=46.42$ mm and depth $d_{c}=32.82$ mm. The moduli of elasticity of the 3D FE model and the springs of the SM are the following: $E_{m}\in \left\{40\times 10^{9},30\times 10^{9},20\times 10^{9},10\times 10^{9},1\times 10^{9},1\times 10^{8},1\times 10^{7},1\times 10^{6}\right\}$ Pa for the matrix, and $E_{p}=40\times 10^{9}$ Pa for the particles. Poisson’s ratio of the particles and matrix for the 3D FE model are $\nu_{p},\nu_{m}\in \left\{0.0,0.33\right\}$, and the Poisson’s ratios for the particle and matrix are the same, i.e., $\nu_{p}=\nu_{m}$. The stiffnesses of the springs were calculated by Equations (9) and (10). The elastic constants of the springs for SM were taken as for the uniaxial stress state, i.e., $D_{i}=E_{i}$, i∈{p,m}. Therefore, Poisson’s ratio does not affect the stiffnesses $K_{s,I}$ of the springs. The length of the connecting elements $L_{c}+d_{p}$ and the distance between the particles $L_{p}$ of the samples depend on the volume fraction $\phi_{p}$: $L_{c}=3.4$ mm for $\phi_{p}=28.46\%$ ($d_{p}=10$ mm), $L_{c}=1.4$ mm for $\phi_{p}=51.45\%$ ($d_{p}=12$ mm) and $L_{c}=0.54$ mm for $\phi_{p}=61.35\%$ ($d_{p}=13$ mm). It is determined that the stiffness $K_{s,FEM}$ is closer to $K_{s,I}$ than to $K_{s,II}$ as $L_{c}$ is small enough. Therefore, the results calculated only with the stiffness $K_{s,I}$ are presented hereafter. The 3D FE model and SM of the composite consist of tetrahedron lattices.

**Figure 7:**
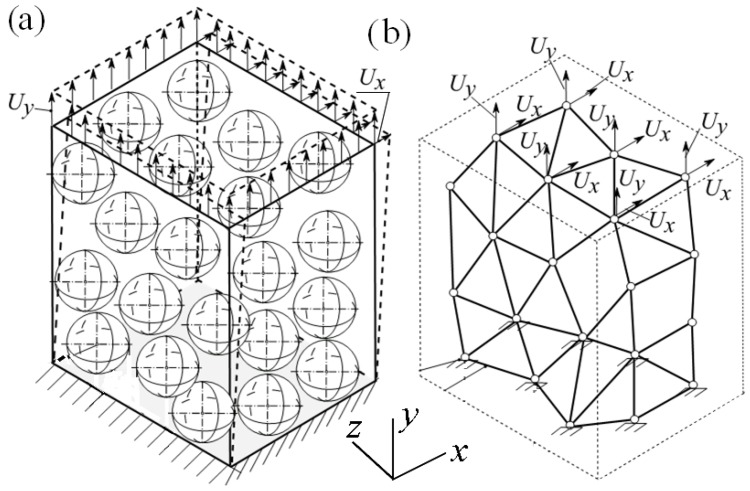
Tensile and shear displacements applied to the samples: 3D FE model of the cube (**a**) and a visualization of the spring model (**b**).

To validate the proposed methodology, the vertical $U_{y}$ and horizontal $U_{x}$ displacements in the directions *y* and *x* were imposed to the top planes of the corresponding samples, see Figure 7, and the tensile $F_{y,3D}$ and shear $F_{x,3D}$ forces of the 3D FE model were compared with the corresponding tensile $F_{y,1D}$ and shear $F_{x,1D}$ forces of the FE model of SM. The boundary conditions for the 3D FE model and SM were as follows: the displacements of the bottom plane of the 3D FE model and SM were restricted fully, i.e., $U_{x}=U_{y}=U_{z}=0$, see Figure 7.

Two loading cases were applied to the specimens to validate the proposed methodology, see Table 1.

**Figure 8:**
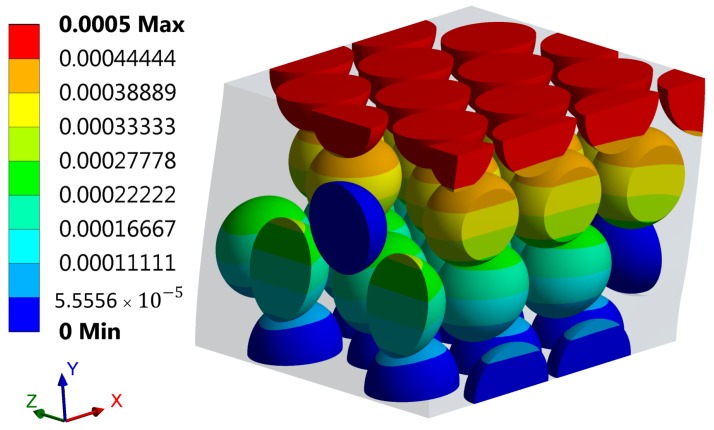
Shear displacements $U_{x}$ (in m) of the 3D-FE model of the cube in direction *x* subject to loading case LC2.

For the sake of illustration, the shear displacements $U_{x}$ of the 3D FE model in the direction *x* subject to loading case LC2 are shown in Figure 8.

**Figure 9:**
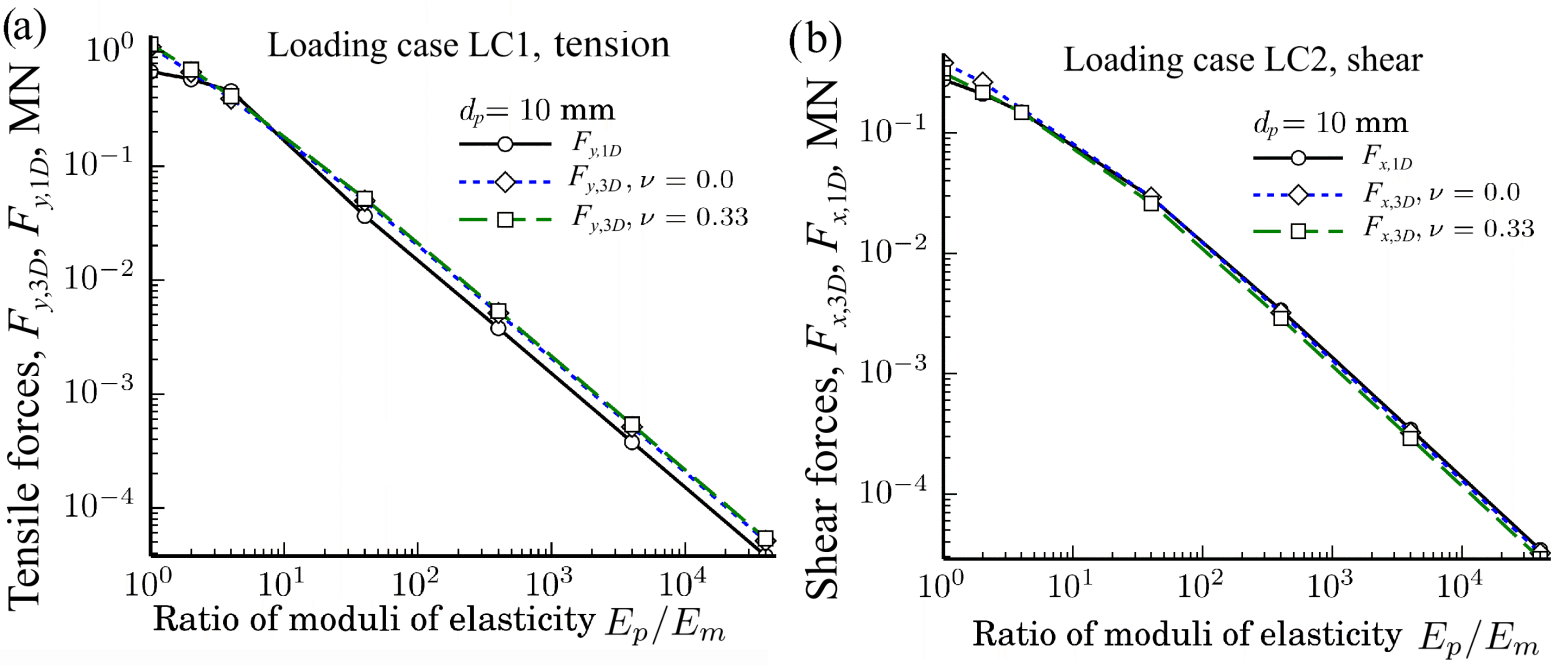
Dependences of the tensile forces $F_{y,3D}$ and $F_{y,1D}$ for (**a**); and the shear forces $F_{x,3D}$ and $F_{x,1D}$ for (**b**) of the loading cases LC1 and LC2 on the ratio $E_{p}/E_{m}$ at different Poisson’s rations $\nu_{p}\in \left\{0.0,0.33\right\}$ when particles’ diameters $d_{p}=10$ mm.

The dependences of the tensile and shear forces $F_{y,1D}$, $F_{y,3D}$, $F_{x,1D}$, and $F_{x,3D}$, of the loading cases LC1 and LC2 on the ratio $E_{m}/E_{p}$ calculated by the 3D FE method and by SM, when $d_{p}=10$ mm at different Poisson’s ratios $\nu_{p},\nu_{m}\in \left\{0.0,0.33\right\}$ and different particles diameters $d_{p}\in \left\{13,12,10\right\}$ mm in double logarithmic scales, are shown in Figure 9. It should be noticed that the calculated tensile and shear forces $F_{y,1D}$ and $F_{x,1D}$ of SM do not depend on Poisson’s ratios $\nu_{p},\nu_{m}\in \left\{0.0,0.33\right\}$, since the calculations were performed as $D_{i}=E_{i}$, i∈{p,m}, i.e., $D_{i}$ does not depend on Poisson’s ratio.

As we can see from Figure 9, the agreement between the results of $F_{x,1D}$ and $F_{x,3D}$ as well as between $F_{y,1D}$ and $F_{y,3D}$ seems very good in double logarithmic scale at various ratios $E_{p}/E_{b}$ and ν∈{0.0,0.33}. However, the relative ratios of the forces $\left(F_{y,3D}-F_{y,1D}\right)/F_{y,3D}$ and $\left(F_{x,3D}-F_{x,1D}\right)/F_{x,3D}$ can reveal the agreement between the results better.

**Figure 10:**
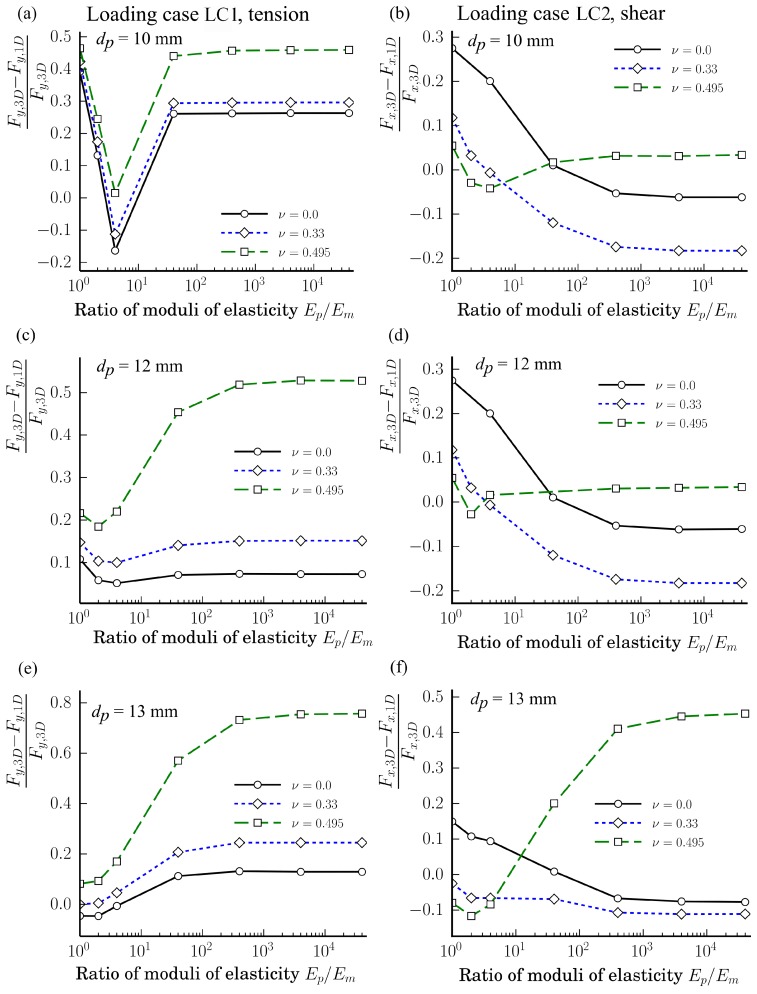
Dependences of the relative ratios $\left(F_{y,3D}-F_{y,1D}\right)/F_{y,3D}$ and $\left(F_{x,3D}-F_{x,1D}\right)/F_{x,3D}$ of the axial and shear forces of loading cases LC1 and LC2 on the ratio $E_{p}/E_{m}$ at different Poisson’s rations $\nu_{p}\in \left\{0.0,0.33,0.495\right\}$ and particles diameters $d_{p}\in \left\{13,12,10\right\}$ mm: (**a**,**b**) as $d_{p}=13$ mm; (**c**,**d**) as $d_{p}=12$ mm; (**e**,**f**) as $d_{p}=10$ mm.

The dependences of the relative ratios $\left(F_{y,3D}-F_{y,1D}\right)/F_{y,3D}$ and $\left(F_{x,3D}-F_{x,1D}\right)/F_{x,3D}$ of the loading cases LC1 and LC2 on the ratio $E_{p}/E_{m}$ at different Poisson’s rations $\nu_{p}\in \left\{0.0,0.33,0.495\right\}$ and particle diameters $d_{p}\in \left\{13,12,10\right\}$ in semi-logarithmic scales are shown in Figure 10. The relative ratios of loading case LC1 are shown in Figure 10a,c,e while those for the loading case LC1 are shown in Figure 10b,d,f. The ratios shown in Figure 10a,b correspond to the case when $d_{p}=13$ mm, whereas, in (c) and (d), to the case when $d_{p}=13$ mm, and in (e) and (f) to the case when $d_{p}=10$ mm.

As we can see from Figure 10, there is not any unique tendency for the ratios $|\left(F_{x,3D}-F_{x,1D}\right)/F_{x,3D}|$ and $|\left(F_{y,3D}-F_{y,1D}\right)/F_{y,3D}|$ dependent on $E_{p}/E_{m}$ except for the fact that the variation of the relative ratios is smaller when $E_{p}/E_{m}>10^{2}$ Pa. From Figure 10, we can also see that for the tension loading case LC1, when $d_{p}\in \left\{12,13\right\}$ mm, the relative difference $|\left(F_{y,3D}-F_{y,1D}\right)/F_{y,3D}|$ is the biggest as $\nu_{p}=\nu_{m}=0.495$. However, a similar conclusion is not valid for the shear loading case LC2. Only when $d_{p}=13$ mm and $E_{p}/E_{m}\geq 40$, the ratio $|\left(F_{y,3D}-F_{y,1D}\right)/F_{y,3D}|$ is the biggest for $\nu_{m}=\nu_{p}=0.495$. The value of the Poisson ratio 0.495 is an extreme case. It real life, for common materials, the Poisson ratio can be assumed as ν∈[0.15,0.40]. The analysis showed that, for the calculated cases, the following limits are valid as $d_{p}\in \left\{10,12,13\right\}$ mm: for the loading case LC1 $\left(F_{y,3D}-F_{y,1D}\right)/F_{y,3D}\in \left[-0.16,0.39\right]$ as ν=0.0 and $\left(F_{y,3D}-F_{y,1D}\right)/F_{y,3D}\in \left[-0.11,0.42\right]$ as ν=0.33; while for the loading case LC2 $\left(F_{x,3D}-F_{x,1D}\right)/F_{x,3D}\in \left[-0.08,0.27\right]$ as ν=0.0, and $\left(F_{x,3D}-F_{x,1D}\right)/F_{x,3D}\in \left[-0.18,0.12\right]$ as ν=0.33. Since the width of the intervals of the relative ratios of the loading case LC2 are less than for the loading case LC1, then the proposed methodology is more accurate for LC2 than for LC1.

**Figure 11:**
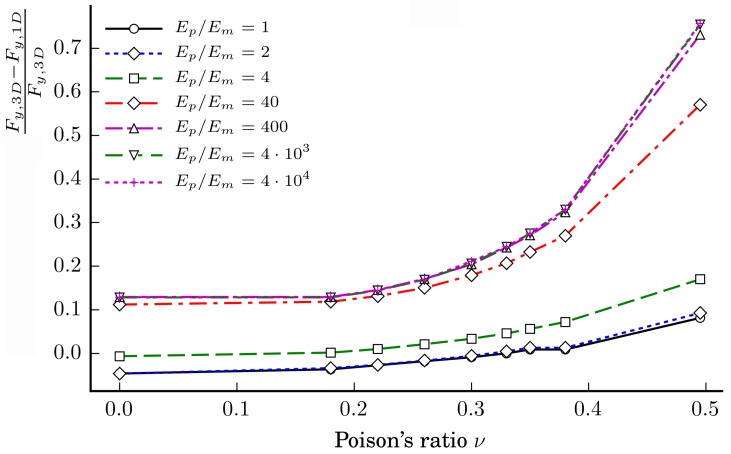
Dependences of the relative ratios $\left(F_{y,3D}-F_{y,1D}\right)/F_{y,3D}$ of the axial forces of the loading case LC1 on the Poisson ratio $\nu_{p}=\nu_{m}$ at different ratios $E_{p}/E_{m}$ when particles’ diameter $d_{p}=13$ mm.

The dependencies of the relative ratios $\left(F_{y,3D}-F_{y,1D}\right)/F_{y,3D}$ of the axial forces of the loading case LC1 on the Poisson ratios $\nu_{p}=\nu_{m}\in \left\{0.0,0.18,0.22,0.26,0.3,0.33,0.35,0.38,0.495\right\}$ at different ratios $E_{p}/E_{m}\in \left\{1,2,4,40,400,4\times 10^{3},4\times 10^{4}\right\}$ when the particles’ diameter $d_{p}=13$ mm are shown in Figure 11.

The figure clearly shows that, when $D_{P}=13$ mm, the relative ratio $\left(F_{y,3D}-F_{y,1D}\right)/F_{y,3D}$ increases with increasing the ratio $E_{p}/E_{m}$. In addition, the relative ratio increases with increasing the Poisson ratios $\nu_{p}$ and $\nu_{m}$ of the particles and matrix, respectively. The relative ratio increases relatively slowly within the interval $\nu_{m},\nu_{p}\in \left[0.0,0.2\right]$ and sharply when $\nu_{m},\nu_{p}\leq 0.38$.

The obtained relative ratios may be treated as too big; however, the effective mechanical properties have to know to approximate a particulate composite as a homogeneous solid by the springs. This prognosis is always inaccurate due to many factors affecting the properties of a composite that cannot be taken into account in the calculations. For example, the well known Hashin–Shtrikman lower and upper bounds [14] may also vary within wide intervals.

When Poisson’s ratio $\nu_{p}=\nu_{m}=0$, then the obtained axial forces $F_{y,3D}$ of the 3D FE model of the loading case LC1 can be compared with the axial forces $F_{y,HS,low}=U_{y}E_{eff,low}A/h_{c}$ and $F_{y,HS,up}=U_{y}E_{eff,up}A/h_{c}$ of the homogeneous cube whose effective elastic moduli $E_{eff,low}$ and $E_{eff,up}$ are calculated by Hashin–Shtrikman’s bounds, where $A=b_{c}d_{c}$ is the cross-section area of the cube. The dimensions of the homogeneous cube are the same as for the 3D FE model shown in Figure 6. The moduli $E_{eff,low}$ and $E_{eff,up}$ are calculated by taking into account the volume fractions of the particles $\phi_{p}$ of the 3DFE model: $\phi_{p}=28.46\%$ as $d_{p}=10$ mm, $\phi_{p}=51.45\%$ as $d_{p}=12$ mm, and $\phi_{p}=61.35\%$ as $d_{p}=13$ mm. The analysis has shown that $F_{y,HS,low}$ is closer to $F_{y,3D}$ than $F_{y,HS,up}$ to $F_{y,3D}$. It is also determined that the relative ratios $|1-F_{y,HS,low}/F_{y,3D}|<|1-F_{y,1D}/F_{y,3D}|$ when $\phi_{p}\in \left\{28.46,51.45\right\}\%$ or when $d_{p}\in \left\{10,12\right\}$ mm; however, when $\phi_{p}=61.35\%$ or $d_{p}=13$ mm, then $|1-F_{y,HS,low}/F_{y,3D}|>|1-F_{y,1D}/F_{y,3D}|$. Therefore, when the distance between the particles’ surfaces decrease, the efficiency of the proposed methodology increases in comparison to the Hashin–Shtrikman bounds. It should be noted that the already existing methodologies for predicting the effective mechanical properties of composites cannot be applied directly to the lattice model, since the stiffnesses of the connecting element are to be determined. The extra methodology has to be involved in the calculations. These considerations show that the proposed methodology can be useful for predicting the stiffness constants of the connecting element. Moreover, as the analysis showed, the greater the ratio $E_{p}/E_{m}$ is, the more accurate is the proposed methodology in comparison to well-known methodologies of the prediction of the effective mechanical properties of the composites due to the fact that the relative ratios $F_{i,3D}$ and $F_{i,1D}$, i∈{x,y} do not increase very much with increasing the ratio $E_{p}/E_{m}$.

## 3. Conclusions

The evaluation of the axial stiffness of the springs for elastic particulate composites of spherical particles is the main subject of the present article. The methodology takes into account spherical surfaces of the particles and the bond. The closed-form solutions of two upper and lower bounds of stiffness have been obtained.

The obtained formulas have been verified by the three-dimensional FE model of the connecting element. In the majority of cases, the stiffness of the connecting element obtained by the three-dimensional FE model is between the lower and upper bounds. In the analysis, it has also been shown that the lower limit of the stiffness is closer to the results obtained by the three-dimensional FE method than the upper limit when the distance between the particle surfaces is small.

The spring model has been verified by a three-dimensional FE model of the elastic particulate composite cube subject to the tension and the shear force when the lower bound of the axial stiffness of the springs was taken into the calculations. It has been shown in the analysis that the absolute values of the relative ratios of the tensile and shear forces of the spring model and the three-dimensional FE models may reach even up to 42%. However, the prognosis of the effective mechanical properties of the composites using the existing methodologies is almost always inaccurate.. Therefore, the proposed methodology of the evaluation of stiffness of the springs has acceptably high accuracy and can be applied to the spring method of particulate composites. The proposed methodology can also be suitable to evaluate the stiffness of the springs of particulate composites of bonded particles when the diameter of the bond is the same as the diameter of the particles.

## Figures and Tables

**Table 1:** Loading cases for the 3D and spring method models.

Loading Case	Displacement $U_{x}$	Displacement $U_{y}$
LC1	-	0.5 mm
LC2	0.5 mm	-

## Abbreviations

The following abbreviations and main notations are used in this manuscript:

$D_{p}$ and $D_{m}$	are elastic constants of the particle and interface member (matrix), respectively
$E_{p}$ and $E_{m}$	are moduli of elasticity of the particle and interface member (matrix), respectively
$F_{x,1D}$ and $F_{y,1D}$	are shear (in direction *x*) and normal (in direction *y*) forces of the the spring model of the particulate composite cube
$F_{x,3D}$ and $F_{y,3D}$	are shear (in direction *x*) and normal (in direction *y*) forces of the the 3D FE model of the particulate composite cube
$K_{s}$	is stiffness of a spring or a connecting element
$K_{s,I}$ and $K_{s,II}$	are limit axial stiffnesses of the spring
$K_{s,FEM}$	is stiffness of the connecting element obtained by the FE model
LC1, LC2	are loading cases
$L_{c}$	is distance between particles’ surfaces
SM	is a spring model consisting of pin-connected springs
$U_{x}$ and $U_{y}$	are displacements in directions *x* and *y*, respectively
$R_{p}$ and $d_{p}$	are radius and diameter of a particle
$R_{b}$	is radius of the interface member
$\nu_{p}$ and $\nu_{m}$	are Poisson’s ratios of the particle and interface member (matrix), respectively
$\phi_{p}$	is particle volume fraction of a composite

## References

[1] Jagota A., Bennison S. (1994). Spring-network and finite-element models for elasticity and fracture. Non-Linearity and Breakdown in Soft Condensed Matter.

[2] Ostoja-Starzewski M. (2002). Lattice models in micromechanics. Appl. Mech. Rev..

[3] Bosia F., Merlino M., Pugno N.M. (2015). Fatigue of self-healing hierarchical soft nanomaterials: The case study of the tendon in sportsmen. J. Mater. Res..

[4] Pal R.K., Ruzzene M., Rimoli J.J. (2016). A continuum model for nonlinear lattices under large deformations. Int. J. Solids Struct..

[5] Wang G., Al-Ostaz A., Cheng A.D. (2009). Mantena, P. Hybrid lattice particle modeling: Theoretical considerations for a 2D elastic spring network for dynamic fracture simulations. Comput. Mater. Sci..

[6] Chen H., Meng L., Chen S., Jiao Y., Liu Y. (2016). Numerical investigation of microstructure effect on mechanical properties of bi-continuous and particulate reinforced composite materials. Comput. Mater. Sci..

[7] Dosta M., Dale S., Antonyuk S., Wassgren C., Heinrich S., Litster J.D. (2016). Numerical and experimental analysis of influence of granule microstructure on its compression breakage. Powder Technol..

[8] Jiang M., He J., Wang J., Zhou Y., Zhu F. (2017). Discrete element analysis of the mechanical properties of deep-sea methane hydrate-bearing soils considering interparticle bond thickness. Comptes Rendus Mécanique.

[9] Peng Y., Sun L.J. (2016). Micromechanics-based analysis of the effect of aggregate homogeneity on the uniaxial penetration test of asphalt mixtures. J. Mater. Civ. Eng..

[10] Schlangen E., Garboczi E. (1997). Fracture simulations of concrete using lattice models: Computational aspects. Eng. Fract. Mech..

[11] Peng J., Wong L.N.Y., Teh C.I. (2017). Effects of grain size-to-particle size ratio on micro-cracking behavior using a bonded-particle grain-based model. Int. J. Rock Mech. Min. Sci..

[12] Potyondy D., Cundall P. (2004). A bonded-particle model for rock. Int. J. Rock Mech. Min. Sci..

[13] Zabulionis D., Kačianauskas R., Rimša V., Rojek J., Pilkavičius S. (2015). Spring method for modelling of particulate solid composed of spherical particles and weak matrix. Arch. Civ. Mech. Eng..

[14] Hashin Z., Shtrikman S. (1963). A variational approach to the theory of the elastic behaviour of multiphase materials. J. Mech. Phys. Solids.

